# The *Drosophila Him* gene is essential for adult muscle function and muscle stem cell maintenance

**DOI:** 10.1016/j.isci.2026.114670

**Published:** 2026-01-10

**Authors:** Robert Mitchell-Gee, Robert Hoff, Kumar Vishal, Daniel Hancock, Sam McKitrick, Cristina V. Newnes-Querejeta, Antonio Aguayo, David Liotta, Jennifer A. Waters, TyAnna L. Lovato, Richard M. Cripps, Michael V. Taylor

**Affiliations:** 1 School of Biosciences, Cardiff University, Cardiff CF10 3AX, UK; 2 Department of Biology, San Diego State University, San Diego, CA 92182, USA; 3 Department of Biological Sciences, San Jose State University, San Jose, CA 95192, USA; 4 Department of Biology, University of New Mexico, Albuquerque, NM 87131, USA

**Keywords:** genetics, cell biology

## Abstract

Muscle stem cells (MuSCs), or “satellite cells,” are vital for vertebrate muscle growth, homeostasis, and repair. The discovery of analogous cells in *Drosophila* opens experimental opportunities in this genetically tractable model. Here, we show that the myogenic inhibitor gene *Him*, as well as being expressed in the myoblasts that form the flight and jump muscles, is expressed in flight muscle MuSCs. This makes *Him* only the second marker of these insect adult MuSCs. Furthermore, *Him* mutants exhibit disrupted jump muscle organization, impaired jumping ability, and a reduced pool of flight muscle myoblasts. In the flight muscles themselves, *Him* mutants show an age-dependent decrease in MuSC number, indicating *Him* is required for MuSC maintenance. This decrease coincides with reduced flight performance. Thus, *Him* is a new marker of *Drosophila* adult MuSCs and is the first gene shown to be required as flies age to maintain both MuSC number and flight ability.

## Highlights

• The *Him* gene is second marker for muscle stem cells (MuSCs) in adult flight muscle
• *Him* mutants have defective adult jump muscle structure and function
• *Him* mutants undergo an age-dependent decrease in MuSC number
• *Him* mutants show an age-dependent decline in flight ability

## Graphical abstract

**Figure:**
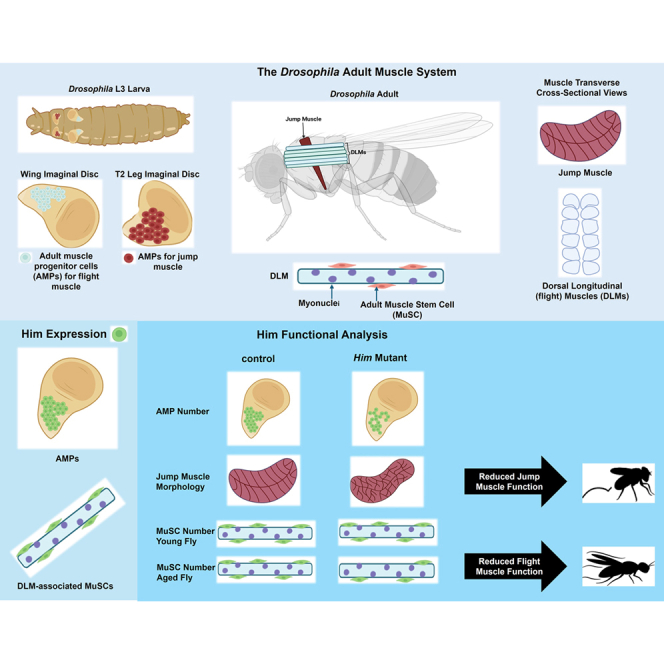

## Introduction

The fruit fly *Drosophila melanogaster* has proven to be a valuable model for researchers to explore the genetic and cellular basis of muscle development.^1^^,^^2^ During development, *Drosophila* undergoes two waves of skeletal muscle myogenesis: the first, during embryogenesis, gives rise to the larval musculature that is used until pupation; the second wave, during pupation, forms the diverse array of muscles found in the adult fly that function for up to 3 months. The different adult muscles arise from adult muscle progenitor cells (AMPs), a stem cell population that is put aside during embryogenesis and which then proliferates during larval life.^3^^,^^4^^,^^5^ The adult muscles include the thoracic indirect flight muscles (IFMs), which develop from wing imaginal disc-associated AMPs, and the jump muscle (also known as TDT, tergal depressor of trochanter), which develops from AMPs associated with the T2 mesothoracic leg imaginal disc.^6^

In recent years, muscle progenitor cells have been discovered in *Drosophila* at the periphery of the dorsal longitudinal muscles (DLMs) of the indirect flight musculature.^7^^,^^8^ These are the adult muscle stem cells (MuSCs), also referred to as “satellite cells,” due to their position at the outer edge of the muscle fiber. Here we shall refer to them as adult MuSCs. In mammals, these cells have been characterized as individual resident adult stem cells that lie quiescent under the basal lamina, and are required for muscle homeostasis and repair after injury.^9^^,^^10^^,^^11^ Although first observed in vertebrates in electron microscopy studies as long ago as 1961, adult MuSCs had not been thought to be present in *Drosophila* muscle. Now three papers have established their credentials. As well as occupying a comparable position on the periphery of the adult muscle fiber, lineage tracing has evidenced that they contribute to the flight muscles in both homeostasis and after injury,^7^^,^^8^ and there is conserved function of the *Rack1* gene in muscle repair.^12^ These *Drosophila* adult MuSCs express the transcription factor Zfh1, whose product is used as a marker of *Drosophila* MuSCs. In mice, the mammalian ortholog of Zfh1, named ZEB1, is also expressed in their MuSCs.^13^

A subtractive hybridization screen for genes expressed in mesoderm derivatives^14^ identified the *Him* gene. In the embryo, *Him* is expressed in muscle progenitor cells, but expression rapidly decreases as muscle differentiation gets underway.^15^^,^^16^ During adult myogenesis, *Him* is expressed in the wing imaginal disc AMP cell population, before downregulation during differentiation.^17^ Functionally, *Him* can inhibit muscle differentiation in both phases of myogenesis.^15^^,^^17^ The *Him* protein binds to the conserved transcriptional co-repressor Groucho, via a C-terminal tetrapeptide WRPW, and requires WRPW and *groucho* function to inhibit muscle differentiation.^15^ Moreover, both in tissue culture and *in vivo*, *Him* can inhibit the activity of the Mef2 transcription factor protein,^15^ a key regulator of muscle differentiation.^18^ These characteristics, together with *Him*’s striking expression in embryonic and adult muscle progenitors and subsequent downregulation during differentiation, suggest a possible role for *Him* in regulating muscle development.

Here, using two new *Him* null alleles, we have uncovered roles for *Him* in both the development and maintenance of adult muscle. *Him* loss of function causes both a reduction in the number of wing-disc associated AMPs as well as an age-associated decline in MuSCs in the adult flies, with a concomitant functional effect. Furthermore, we show a requirement for *Him* in normal jump muscle development and function.

## Results and discussion

### *Him* is a new marker of *Drosophila* adult MuSCs

**Figure 1:**
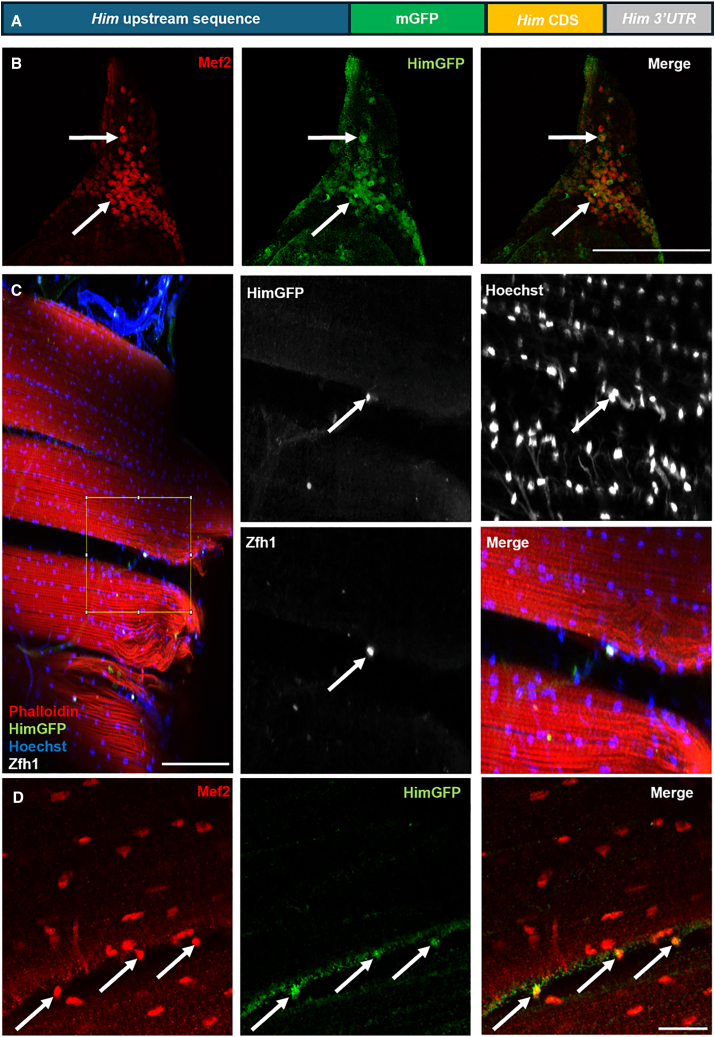
HimGFP design and expression in leg disc myoblasts and adult MuSCs (A) Schematic of the HimGFP “minigene” comprising 3.8 kb upstream genomic sequence, mGFP fused to the *Him* coding sequence via an -SSSS- linker, followed by the *Him* 3′ UTR. (B) Larval T2 leg discs labeled with anti-Mef2 and anti-GFP show co-expression of HimGFP and Mef2 (examples arrowed) in the associated myoblasts (*n* = 4). Scale bars, 100 μm. (C) Sagittal thoracic section showing an adult MuSC (arrows) on the periphery of a DLM fiber (boxed area shown at higher magnification). The MuSC expresses HimGFP and canonical marker Zfh1, and stains with nuclear marker Hoechst (*n* = 5). Scale bars, 100 μm. (D) Close-up of a sagittal thoracic section showing adult MuSCs (arrowed) on the periphery of a DLM fiber that express both HimGFP and Mef2 (*n* = 3). Scale bars, 20 μm.

To visualize *Him* expression during development, we used a HimGFP “minigene” (Figure 1A). This minigene comprises approximately 3.8 kb of upstream *Him* sequence, followed by N-terminally mGFP-tagged Him coding sequence and the *Him* 3′UTR.^15^ This minigene has previously been shown to be co-expressed with Mef2 in the AMPs associated with the wing imaginal disc.^17^ We now show that *Him* is also expressed in the equivalent cells in the T2 leg imaginal disc, that is, the jump muscle progenitor cells, and is again co-expressed with Mef2 (Figure 1B). Thus, *Him* expression is a marker of the AMPs (myoblasts) during the larval stage.

*Him* expression is known to decline during adult muscle development,^17^ but we observed a small number of isolated cells expressing HimGFP on the periphery of the adult DLM fibers. Figure 1C shows these cells co-express HimGFP and the canonical adult MuSC marker Zfh1.^7^^,^^8^ Furthermore, these HimGFP-positive cells are also positive for the muscle lineage marker, Mef2 (Figure 1D). This result is supplemented by showing that these cells are negative for a hemocyte marker, Pxn (Figure S1A). The characterization of Mef2-positive, hemocyte marker-negative has been used previously to support the classification of these cells as MuSCs.^8^ The *Him* expression result is additionally supplemented by showing that HimGal4-driven UAS-mCherry expression co-localizes with Zfh1 in cells at the periphery of the DLM fiber (Figure S1B). Thus, *Him* is a marker of the *Drosophila* adult MuSCs and only the second such marker to be described.

### Generation of *Him* mutants

**Figure 2:**
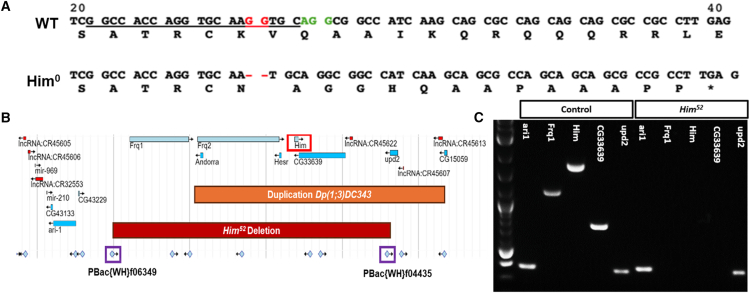
Generation of *Him* mutants (A) Partial wild-type (WT) *Him* versus *Him*^*0*^ coding sequence before and after a CRISPR-mediated 2 nucleotide deletion between codons 25–26. WT sequence shows protospacer (underlined), PAM (green text) and the two nucleotides lost in the *Him*^*0*^ mutant (red). Translations below coding sequences show the 2-nt deletion causes a premature stop (∗). (B) JBrowse (FlyBase) map of the *Him* locus (red box) showing the *Dp(1;3)DC343* duplication (orange box), which includes 81,651bp DNA spanning *Frq2*, *Andorra*, *Hesr*, *Him*, *CG33639* and *upd2*, and the two pBacWH elements (purple boxes) used to generate the 98 kb *Him*^*52*^ deletion. (C) PCR analysis of *Him*^*52*^ deletion gDNA shows amplicons outside of the deletion (*ari1* and *upd2*) but not within it (*Frq1*, *Him*, *CG33639*). All 5 amplicons are in the control.

We next wished to determine if *Him* has functions in the cells or tissues linked to these areas of expression. To do this, we made two new mutant alleles of the *Him* gene (Figure 2). First, *Him*^*0*^ was generated using CRISPR-targeting of the *Him* gene (Figure 2A). We isolated an allele with a 2-nucleotide deletion near the 5′ end of the *Him* coding sequence, at codon 25. This produced a frameshift and resulted in a premature stop codon at codon 39 and a presumed null allele. Second, *Him*^*52*^ was made by transposable element mediated *trans*-recombination between two pBac elements, *pBac{WH}f06349* and *pBac{WH}f04435* (Figure 2B). This produced an approximately 98 kb deletion that includes *Him* plus five other genes. This was verified by PCR: genes between the pBac elements (*Frq1*, *Him*, and *CG33639*) cannot be PCR amplified from *Him*^*52*^ genomic DNA unlike from control DNA, whereas genes distal (*ari1*) and proximal (*upd2*) to the pBac elements can be amplified from both *Him*^*52*^ and control (Figure 2C). Both the *Him*^*0*^ and *Him*^*52*^ alleles are viable. For the rescue experiments in our genetic characterization of the *in vivo* function of *Him*, we also used the previously published HimGFP minigene^15^ and an available duplication of the *Him* chromosomal region (*Dp(1;3)DC*343). Thus, taken together, we have the tools for analyzing the role of *Him* in myogenesis.

### *Him* is required for normal jump muscle morphology

The jump muscle is a tubular muscle in the adult thorax, and comprises two columns of muscle fibers neatly organized in an ovoid pattern around a mid-line with each fiber contacting the mid-line and outer surface of the muscle. These fibers stretch from the mesothoracic leg (T2) to the dorsal notum of the thorax and contract to power the force required for the insect to jump.^19^ The arrangement is stereotypical, although the number of fibers that make up the jump muscle vary depending on the wild-type (WT) strain.^6^

**Figure 3:**
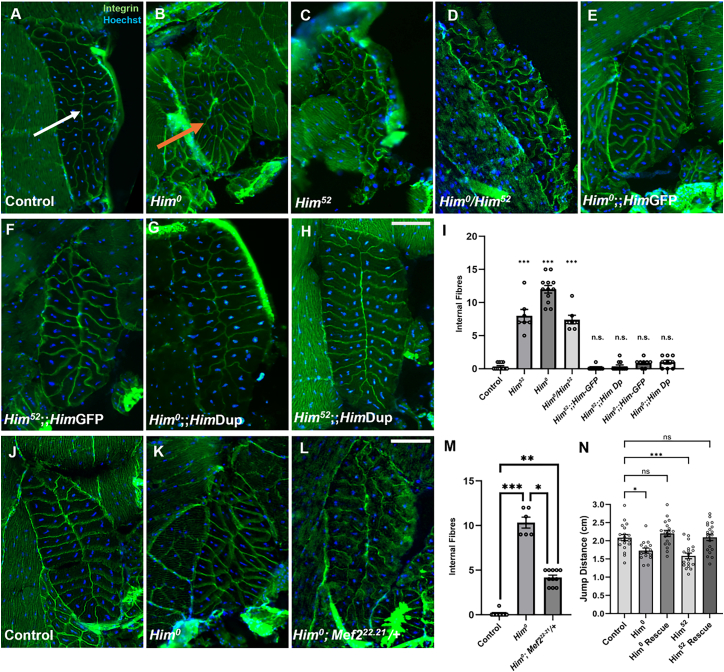
*Him* is required for normal jump muscle morphology and function, and genetically interacts with Mef2 (A–H) Transverse cryosections of adult jump muscle labeled with anti-βPSintegrin (green) and Hoechst nuclear stain (blue). (A) Control (*yw;;nos-Cas9*) jump muscle section (*n* = 12) consists of two organized rows of muscle fibers arranged around a mid-line (white arrow). The WT morphology is disrupted in 100% of (B) *Him*^*0*^ mutant (*n* = 12), (C) *Him*^*52*^ mutant (*n* = 7), and (D) *Him*^*0*^*/Him*^*52*^ heteroallelic mutant flies (*n* = 7). *Him* mutants have “internal fibers” that do not contact the outer edge of the jump muscle (orange arrow in B). Both the HimGFP minigene and the *Him* duplication restore WT morphology in *Him*^*0*^ (E,G) and *Him*^*52*^ (F,H) mutants (*n* = 8–11 per genotype). Scale bars, 50 μm. (I) Bar graph showing mean internal muscle fiber counts ±SEM of the genotypes in (A–H). *Him* mutants have significantly more internal fibers than WT. Both *Him*GFP and the *Him* duplication restore counts to WT values. (Kruskal-Wallis followed by post hoc Dunn’s test: *p* < 0.001, control versus *Him*^*52*^ ∗∗∗*p* < 0.001, control versus *Him*^*0*^ ∗∗∗*p* < 0.001, control versus *Him*^*52*^/*Him*^*0*^ ∗∗∗*p* < 0.001, control versus *Him*^*52*^;;*Him*GFP *p* = 0.5985 (n.s.), control versus *Him*^*52*^*;*;*Him*Dup *p* = 0.2506 (n.s.), control versus *Him*^*0*^;;*Him*GFP *p* = 0.2784 (n.s.), control versus *Him*^*0*^;;*Him*Dup *n* = 0.2506 (n.s.). (J–L) Transverse cryosections of (J) control (*yw;;nos-Cas9 n* = 11), (K) *Him*^*0*^ mutant (*n* = 6) and (L) *Him*^*0*^*; Mef2*^*22.21*^*/+* flies (*n* = 11). Scale bars, 50 μm. The *Mef2*^*22.21*^ allele rescues the *Him* mutant morphology toward WT. (M) Bar graph showing mean internal muscle fiber counts ±SEM of the genotypes in (K–M). *Him*^*0*^*;Mef2*^*22.21*^*/+* flies have significantly fewer internal fibers than *Him*^*0*^ mutants, but more internal fibers than control flies (Kruskal-Wallis followed by post hoc Dunn’s test: *p* < 0.001, control versus *Him0* ∗∗∗*p* < 0.001, control versus *Him0;Mef2*^*22.21*^*/+* ∗∗*p* = 0.0012, *Him*^*0*^ versus *Him0;Mef2*^*22.21*^/+ ∗*p* = 0.0363). (N) Bar graph showing mean horizontal jump ±SEM, in response to a looming stimulus (*n* = 15–20 flies per genotype). *Him*^*0*^ and *Him*^*52*^ adults jump significantly less distance than *yw;;nosCas9* controls. HimGFP rescues each *Him* mutant to WT phenotype. (ANOVA followed by post hoc Dunnett’s test: ANOVA *p* < 0.0001, control versus *Him*^*0*^ ∗*p* = 0.0151, control versus *Him*^0^;;*Him*GFP/+ non-significant (n.s.) *p* = 0.7185, control versus *Him*^*52*^ ∗∗∗*p* = 0.0001, control versus *Him*^*52*^;*Him*GFP/+ n.s. *p* > 0.9999).

We found that the *Him* mutant alleles had a striking jump muscle morphology phenotype: 100% of flies showed severe disruption to the ordered pattern of fibers. Although the jump muscle still develops, the stereotypical arrangement of the muscle fibers seen in cross-sections is disrupted in both *Him*^*0*^ and *Him*^*52*^ mutants, when compared to control flies (Figures 3A–3D). Instead of two neat columns of muscle fibers, the muscle has a fractured, disorganized appearance with “internal fibers,” defined as fibers not contacting the muscle’s outer surface. This phenotype is fully penetrant, with all *Him* mutant alleles and allelic combinations displaying a dysmorphic jump muscle structure. Thus, *Him*^*0*^ and *Him*^*52*^ look similar. The heteroallelic combination of the CRISPR allele (*Him*^*0*^) with the *Him* mini-deletion (*Him*^*52*^) also has a similar phenotype. Furthermore, both *Him*^*0*^ and *Him*^*52*^ alleles can be rescued to WT morphology by either one copy of the HimGFP minigene or by the *Him* chromosomal region duplication (Figures 3E–3H). All these jump muscle phenotypes were quantified by counts of the number of internal fibers in each condition (Figure 3I).

Taken together, these results show that the *Him* loss-of-function phenotype is not due to any off-target effect in either mutant allele and that the *Him*^*0*^ allele is a null as intended. Moreover, the rescue of *Him* loss of function with the Him-GFP minigene shows that this Him-GFP functions like the WT gene and corroborates its use as a tool for *Him* expression. Thus, we have developed reagents for the analysis of *Him* function and have used them to establish that the *Him* gene is required to make a jump muscle with the normal, ordered arrangement of muscle fibers. Since *Him* has been characterized as an inhibitor of Mef2 activity,^15^^,^^17^ these morphological changes observed in the *Him* mutants may arise from enhanced activity of Mef2. Indeed, *Mef2* over-expression also causes defects in jump muscle patterning.^20^ We therefore tested whether reduced Mef2 activity could suppress this *Him* mutant phenotype. Figures 3J–3M shows that it can. A heterozygous *Mef2* null mutant (*Mef2*^*22.21*^*/+*) rescues the *Him* loss of function jump muscle phenotype toward WT, as assessed by a significant reduction in the number of internal muscle fibers.

### *Him* is required for jump muscle function

To determine if the loss of jump muscle organization has a phenotypic consequence, we undertook a jumping behavioral assay on young adult male flies (<2 days post-eclosion). *Drosophila* jumps both as an escape response from a looming stimulus, or to “take-off” to initiate flight.^21^^,^^22^ Here, we used a paintbrush as the looming stimulus and found both *Him*^*0*^ and *Him*^*52*^ mutant flies had a significant 15%–25% reduction in jumping ability compared to controls (Figure 3N). Similar to the morphological assay, the HimGFP minigene could rescue the behavioral phenotype of both *Him* alleles to WT. This demonstrates a functional consequence of the disrupted muscle morphology. The *Him* gene is therefore required for both jump muscle morphology and function.

### *Him* mutants have a reduced wing disc-associated myoblast pool

**Figure 4:**
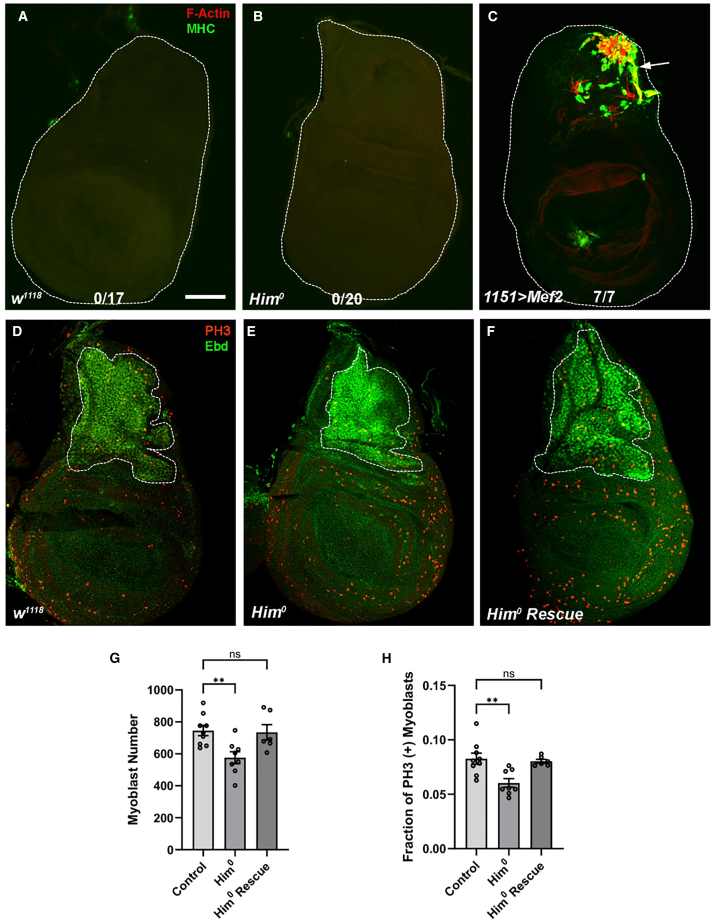
*Him* mutants have a reduced wing disc-associated myoblast pool (A–C) L3 wing imaginal discs were stained for Mhc (green), and F-actin (red). Premature muscle differentiation is not observed in (A) control (*w*^*1118*^) discs (*n* = 17), or (B) *Him*^*0*^ mutants (*n* = 20). (C) 1,151-Gal4-driven UAS-Mef2 was used as a positive control (*n* = 7). Scale bars, 50 μm. (D–F) L3 larval wing imaginal discs were stained for Ebd1 (green), which labels all myoblasts, and phospho-histone H3 (PH3, red), which labels mitotic cells. Scale bars, 50 μm. The myoblast pool size (outlined) was reduced in (E) *Him*^*0*^ mutants (*n* = 8) compared to (D) controls (*n* = 9), and restored (F) when mutants were combined with *Dp(1;3)DC343* (*n* = 6). (G) Quantification of myoblast number for each genotype. (ANOVA followed by post hoc Dunnett’s test: ANOVA *p* = 0.0067, control versus *Him*^*0*^ ∗∗*p* = 0.0064, control versus rescue n.s. *p* = 0.9728). (H) Quantification of proliferating myoblasts shows the fraction of phospho-histone H3 (PH3) expressing cells was reduced in *Him*^*0*^ mutants compared to controls, and restored when mutants were combined with *Dp(1;3)DC343*. (ANOVA followed by post hoc Dunnett’s test: ANOVA *p* = 0.0022, versus control versus *Him*^*0*^ ∗∗*p* = 0.002, control versus rescue n.s. *p* = 0.9064).

We also analyzed the wing imaginal discs of control and *Him* mutant third-instar larvae, to determine if there were any observable consequences of loss of *Him* function. While premature muscle differentiation can be a marker for unrestrained Mef2 activity,^17^^,^^23^ we did not observe F-actin nor myosin accumulation in mutant discs (Figures 4A–4C). However, we did observe a reduced wing disc-associated myoblast pool size in *Him*^*0*^ mutants, and this phenotype was rescued by the *Him Dp(1;3)DC343* duplication (Figures 4D–4F, quantified in Figure 4G). This reduction in the myoblast pool was at least partially caused by a reduction in myoblast proliferation, based upon staining for phospho-histone H3 (Figures 4D–4F, quantified in Figure 4H). Given a role for *Him* in inhibiting myoblast differentiation, reduced proliferation in the *Him* mutants might indicate that the myoblasts are transitioning into a differentiation state.

### *Him* is required to maintain adult MuSC number and flight ability as flies age

**Figure 5:**
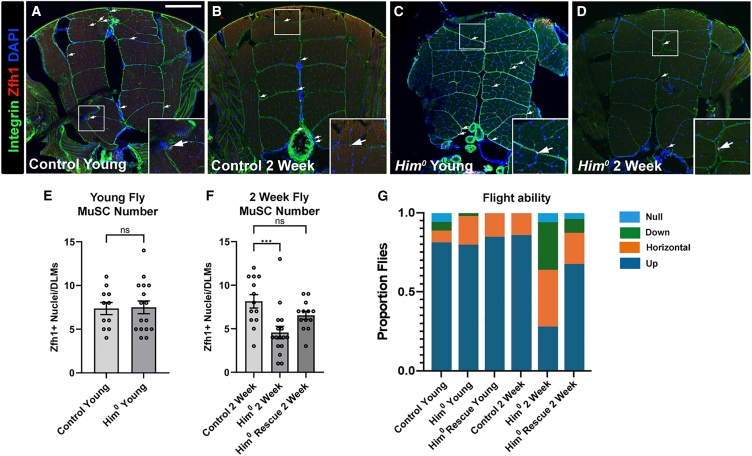
*Him* is required to maintain adult MuSC number and flight ability as flies age (A–D) Representative transverse DLM cryosections of control (*w*^*1118*^) and *Him*^*0*^ mutant flies, for young (<2 days) and 2-week-old flies (*n* = 10–15 flies, per sample). Muscle fibers are outlined using anti-βPSintegrin (green), and MuSCs stained with anti-Zfh1 (arrowed). Scale bar, 100 μm. (E) Bar graph showing mean number of MuSCs ±SEM was similar in young control (*n* = 11) and *Him*^*0*^ mutant (*n* = 16) flies (*t* test n.s., *p* = 0.8985). (F) In flies aged 2 weeks, MuSC number is significantly reduced in *Him*^*0*^ mutants (*n* = 16) compared to controls (*n* = 13) but is restored to WT by combining mutants with *Dp(1;3)DC343* (*n* = 13). (ANOVA followed by post hoc Dunnett’s test: ANOVA *p* = 0.0021, control vs. *Him*^*0*^ ∗∗∗*p* = 0.001, control vs. rescue n.s., *p* = 0.1931). (G) Flies (*n* > 50) of the same genotypes and ages as in (A)–(F) were flight-tested and scored for flying upwards, horizontally, downwards, or not-at-all (null). Controls showed strong flight ability as young flies and when aged; and *Him*^*0*^ mutants showed an age-dependent reduction in flight ability, which was rescued by *Dp(1;3)DC343*. At 2 weeks, *Him*^*0*^ mutant flies were significantly worse at flying than *yw;;nosCas9* controls (*x*^2^ = 30.60, *p* < 0.001).

We then asked whether *Him* function affected the number of adult MuSCs, identified in transverse DLM cross-section by Zfh1 expression and their morphology and location. MuSC nuclei are small and located close to the integrin-containing muscle periphery. In young flies (<2 days), the MuSC number is similar in *Him*^*0*^ mutants as in WT controls (Figures 5A–5C, quantified in Figure 5E). However, after 2 weeks, although the MuSC number in WT flies was unchanged, strikingly in *Him* mutants, we found a significant reduction of approximately 50% in Zfh1-positive MuSC number in DLM cross-sections (Figures 5B–5D, quantified in Figure 5F). This was rescued by the *Him Dp(1;3)DC343* duplication to close to the value in WT flies.

We then asked whether there was any functional consequence to this. Do *Him* mutants show a loss of flight ability over time? We measured flight ability in both young and 2-week-old flies using a standard flight assay that scores whether flies fly upwards, horizontally, downwards or not-at-all, when released below a light in a plexiglass box.^24^ Control *w*^*1118*^ flies predominantly fly upwards, both as young flies and after aging for 2 weeks (>75% in both categories fly upward; Figure 5G). For the *Him*^*0*^ mutants, young adults showed strong flight ability similar to controls (>70% flew upward). However, after 2 weeks, only approximately 25% of *Him*^*0*^ adults showed strong flight, and the rest were moderately to severely impaired in their flight ability. The reduced flight ability phenotype of the *Him*^*0*^ mutant was rescued by the *Him Dp(1;3)DC343* duplication, confirming that this effect is due to mutation of the *Him* gene. Thus, *Him* is required to maintain flight ability as flies age.

Taken together, our studies identify multiple roles for the myogenic inhibitor gene *Him* in muscle development and function. We show that *Him* is required to maintain the MuSC pool both at the larval stage and in the adult, and that in adult *Him* mutants, a loss of MuSCs as flies age is accompanied by an age-dependent reduction in flight ability. Furthermore, *Him* is required for normal jump muscle organization and function. One potential mechanism to explain these phenotypes is that Him can inhibit Mef2 activity.^15^ Mef2 is a driver of muscle differentiation,^18^ whereas genes such as *zfh1* and *Him* can inhibit the myogenic program.^15^^,^^25^ Notably, while Zfh1 levels decline in the majority of wing disc AMPs as development progresses, a subset of cells retains Zfh1 expression and fails to differentiate; these are the MuSCs.^7^^,^^8^ Like Zfh1, Him is prominently expressed in wing disc-derived myoblasts around the DLM templates at about 20 h after pupal formation, as the DLMs begin to develop, but is no longer detected there by 30 h.^7^^,^^17^ It is possible that Him functions alongside Zfh1 to repress the myogenic program. As such, loss of *Him* function could cause enhanced Mef2 activity, which in adult MuSCs might push them toward differentiation. This would be consistent with the observations that enhanced Mef2 function in disc-associated myoblasts phenocopies *Him* loss of function in both interfering with jump muscle organization^20^ and reducing the myoblast pool size.^26^

We also note that Him is only the second identified marker, after Zfh1, for *Drosophila* adult MuSCs. As well as making Him a useful tool for identifying these cells, it provides new knowledge about their biology. Notably, *Him* is the first gene identified that has been demonstrated to be required for maintenance of both adult *Drosophila* MuSCs and flight muscle function. Our studies illustrate the promise of the *Drosophila* system for a genetic dissection of these important cells in the context of the maintenance of developed muscle, as well as of repair following injury, and of muscle deterioration associated with age and disease.

### Limitations of the study

In this characterization of the first null alleles for the *Him* gene, we have focused on several significant aspects of the phenotype linked to *Him* loss of function, although we recognize that other facets await analysis. Also, the cellular and molecular mechanisms that underpin the phenotypes that we have identified are still being uncovered. The genetic interaction of *Him* mutants with a *Mef2* null allele supports earlier studies demonstrating that Him inhibits Mef2 activity, but, here, we have not probed the direct molecular basis for this observation. Similarly, while there is a clear consequence for flight ability from loss of *Him* function as flies get older, further studies will need to be carried out to understand the cellular basis of this reduction in flight ability and its precise relationship to the observed coincident loss of adult MuSCs.

## Resource availability

### Lead contact

Requests for further information and resources should be directed to and will be fulfilled by the lead contact, Michael V. Taylor (taylormv@cardiff.ac.uk).

### Materials availability

Fly stocks generated in this study are available upon request to the lead contact.

### Data and code availability

• Data reported in this paper will be shared by the lead contact upon request.
• This study generated no code.
• Any additional information required to reanalyze the data reported in this paper is available from the lead contact upon request.

## Acknowledgments

Stocks obtained from the 10.13039/100005493Bloomington Drosophila Stock Centre (NIH P40OD018537) were used in this study. We are grateful to Dr. Ruth Lehmann and Dr. Yashi Ahmed for sending antibodies, to the Bioimaging Hub, Cardiff School of Biosciences, and to Brenna Blotz for statistical analysis assistance. This work was supported by GM124498 from the 10.13039/100000052NIH/10.13039/100000057NIGMS to R.M.C. and by the John Ryder Memorial Trust and a 10.13039/501100000268BBSRC PhD studentship to M.V.T.

## Author contributions

Conceptualization, R.M.-G., T.L.L., R.M.C., and M.V.T.; methodology, R.M.-G., R.H., D.H., and C.V.N.-Q.; validation, R.M.-G., R.H., and K.V.; formal analysis, R.M.-G. and R.M.C.; investigation, R.M.-G., R.H., K.V., S.M., C.V.N.-Q., T.L.L., A.A., and J.A.W.; resources, D.H., D.L., and S.M.; writing – original draft preparation, R.M.-G., R.M.C., and M.V.T.; writing – review and editing, M.V.T., R.M.C., and R.M.-G.; visualization, R.M.-G., R.H., and K.V.; supervision, T.L.L., R.M.C., and M.V.T.; project administration, R.M.C. and M.V.T.; fund acquisition, R.M.C. and M.V.T.

## Declaration of interests

The authors declare no competing interests.

## STAR★Methods

### Key resources table

**Table:** 

REAGENT or RESOURCE	SOURCE	IDENTIFIER
**Antibodies**		

Guinea pig anti-Ebd (1:2,000)	Yashi Ahmed	N/A
Rabbit anti-Mef2 (1:1,000)	DSHB	Mef2
Rabbit anti-Zfh1 (1:5,000)	Ruth Lehmann	N/A
Chicken anti-GFP (1:500)	Abcam	Ab13970; RRID: AB_300798
Mouse anti-βPS-Integrin (1:50)	DSHB	CF.6G11
Rabbit anti-PH3 (1:100)	Millipore	H0412
Mouse anti-Mhc (1:100)	DSHB	3E8-3D3
Anti-Chicken-488 (1:500)	Jackson Immuno	703-545-155; RRID: AB_2340375
Anti-Rabbit-555 (1:500)	Thermofisher	A-21428; RRID: AB_2535849
Anti-Rabbit-546 (1:500)	Thermofisher	A-11035; RRID: AB_2534093
Anti-Mouse-488 (1:500)	Thermofisher	A-11029; RRID: AB_2534088

**Chemicals, peptides, and recombinant proteins**		

Hoechst 33258	Merck	94403
Acti-Stain 670 Phalloidin	Cytoskeleton Inc	PHDN1-A
Paraformaldehyde Prilled	Merck	441244
Triton X-100	Severn Biotech	40-2055-05
Phosphate Buffered Saline (PBS) 10X	Severn Biotech	20-7400-10
Bovine Serum Albumin (BSA)	Merck	A9647
Tissue-Tek OCT Compound	Fisher	NC1862249
Glycerol	Merck	G5516

**Critical commercial assays**		

ChargeSwitch DNA extraction Kit	Invitrogen	CS11203

**Experimental models: Organisms/strains**		

*Him*^*52*^	This study	N/A
*Him*^*0*^	This study	N/A
;*HimGFP*	Liotta et al.^15^	N/A
*;;Dp(1;3)DC343*	Bloomington *Drosophila* Stock Centre	BDSC Cat#32277
*yw;;nos-Cas9*	BestGene	N/A
*w*^*1118*^*;;*	Bloomington *Drosophila* Stock Centre	BDSC Cat#3605
*1151Gal4;;*	Anant et al.^27^	N/A
*UAS-Mef2;;*	Cripps et al.^28^	N/A
*;HimGal4;*	Wessel^29^	N/A
*;;UAS-mCherry.NLS*	Bloomington *Drosophila* Stock Centre	BDSC Cat#38424
*;Sco/CyO;Pxn-RFP*	Rodrigues et al.^30^	N/A

**Oligonucleotides**		

*ari1* FWD Primer 5’-CACAAAGGCACAGTCGAAAAGC	N/A	N/A
*ari1* REV Primer 5’- GCACCCATCGATAAGCAGATG	N/A	N/A
*Frq1* FWD Primer 5’-CTTTCCACAAGGCGATCCCA	N/A	N/A
*Frq1* REV Primer 5’-CAGGTTGCCCTTCGATGTG	N/A	N/A
*Him* FWD Primer 5’-AGTTTGGCGCGCAATGTG	N/A	N/A
*Him* REV Primer 5’-GAACGAAGGCAGATGGAG	N/A	N/A
*CG33639* FWD Primer 5’-GGATCTACGTTCATTCATTGTG	N/A	N/A
*CG33639* REV Primer 5’- CGAGGACTAAGTTATCTAGTTACAT	N/A	N/A
*upd2* FWD Primer 5’-CTCGCTATAATACTGGAAGGGC	N/A	N/A
*upd2* REV Primer 5’-GAGCGTCTCAACTTTCACACG	N/A	N/A
*Him*^*0*^ guideRNA oligo 5’-GGCCACCAGGTGCAAGGTGC	N/A	N/A

**Software and algorithms**		

Fiji-ImageJ	ImageJ	https://imagej.net/software/fiji/
R	R	https://www.r-project.org/
Tracker	Tracker	https://physlets.org/tracker/
BioRender	BioRender	https://www.biorender.com/
GraphPad Prism	GraphPad	https://www.graphpad.com/

**Other**		

HM525 NX Cryomicrotome	ThermoScientific	HM525
OTF5000 Cryostat with 5040 microtome	Bright	OTF5000
Olympus BX63 Fluorescent Microscope	Olympus	BX63
Hamamatsu Camera	Hamamatsu	C11440-36U
CoolLED pE-300lite LED lightsource	CoolLite	pE-300lite
Olympus FluoView 3000 Confocal Microscope	Olympus	FluoView 3000
Zeiss LSM880 Confocal Microscope	Zeiss	LSM880

### Experimental model and study participant details

#### Experimental model – *Drosophila melanogaster*

##### Fly husbandry

Fly stocks and experimental crosses were maintained at 25 ͦC on standard culture media. For experiments on larval imaginal discs, unsexed larvae were collected at wandering third instar stage. For imaging of adult MuSCs in sagittal section, female flies were studied as their larger size simplifies the dissection protocol. For assaying *Him* loss-of-function, only males were studied, as in the subsequent rescue experiments only male F1 were of the correct genotype, due to *Him* being located on the X chromosome. The exception to this was the heteroallelic combination of *Him*^*52*^ and *Him*^*0*^*,* which had to be female to carry both X chromosome alleles. Details of fly stocks used can be found in the key resources table.

### Method details

#### Generation of new Drosophila lines

The *Him*^*52*^ line was generated using FRT-mediated recombination between the *pBac{WH}f06349* and *pBac{WH}f04435* elements, resulting in a 98Kb deletion spanning six genes, including *Him*. PCR primer pairs were designed to confirm that the genes between the two pBac elements were deleted, and that genes outside the elements remain intact. Genomic DNA (gDNA) was extracted using Invitrogen’s ChargeSwitch Kit (CS11203), using 3 flies of each genotype. The control flies were *yw;;nos-cas9*. The genes analysed were *ari1, Frq1*, *Him*, *CG33639* and *upd2*. PCR primer sequences can be found in the key resources table.

The *Him*^*0*^ line was generated using CRISPR/Cas9 genome editing. Flies of the genotype *yw*; nos-Cas9 were injected with a sgRNA (IDT) targeting Him (protospacer sequence underlined in Figure 2A). Approximately fifty G0 adults arising from the injected embryos were separately crossed to generate stable lines, and each line was screened for mutations in Him using PCR followed by sequencing. One line, designated Him0, had a 2-nt deletion beginning in codon 25, resulting in a frameshift and subsequent premature stop codon at position 39.

#### Histology

Antibodies and concentrations are reported in the key resources table. All fixation, washing, blocking and staining steps were performed at room temperature with agitation on a rotary mixer, unless otherwise specified.

#### Immunostaining: Imaginal discs

Wandering 3^rd^ instar larvae were dissected in Phosphate Buffered Saline (PBS), and fixed in 4% paraformaldehyde (PFA) for 20 minutes. Samples were then rinsed quickly 3x using PBS containing 0.03% Triton X-100 (PBT), followed by 3x10 minute washes in 0.03% PBT. Blocking was performed in 0.03% PBT containing 0.5% bovine serum albumin (BSA), for 30 minutes. Samples were incubated with primary antibodies overnight at 4 ͦC on a rotary mixer. Samples were then rinsed and washed (3x10 minutes) in 0.03% PBT, before incubation with secondary antibodies for 2 hours. Dapi and phalloidin were included with secondary antibodies when appropriate, at a concentration 1:3,000, or 100nM respectively. Samples were rinsed and washed (3x 10 minutes) in 0.03% PBT, before mounting in 80% glycerol in PBS.

#### Immunostaining: Cryosections of adult muscles

Cryosectioning was used to visualize transverse views of the jump muscle for phenotyping of *Him* mutants, and transverse views of the DLMs for quantification of MuSCs. Jump muscle cryosections were of young adult flies (<2 days). DLM cryosections were of young adult flies (<2 days), or flies aged for 2 weeks.

Whole flies were mounted dorsal side down (jump muscle sectioning), or thoraces were mounted anterior side down (DLM sectioning) on double sided tape. Samples were then submerged in OCT before being snap frozen in liquid nitrogen, and stored at -80 ͦC until sectioning. Samples were transferred to a cryostat at -30 ͦC, where serial 10-12 μm sections were generated and collected on microscope slides.

Slides were transferred to a 100ml slide holder, and fixed for 8 minutes in 4% PFA. The fixative was removed, and slides were washed with 0.1% PBT for 3 minutes, followed by a 30 minute wash, followed by 3x5 minute washes. Samples were blocked with 0.5% BSA/0.1% PBT for 30 minutes. 100μl primary antibody, diluted in 0.5%BSA/0.1% PBT, was added to each slide, followed by a cover slip. Slides were incubated overnight in darkness in a humidity chamber at room temperature. Slides were washed for 5 minutes with 0.1% PBT, before incubation with secondary antibody diluted in 0.5% BSA/0.1% PBT for 2 hours. A final 5 minute wash was performed with 0.1% PBT, before mounting the samples in 50% glycerol diluted in PBS.

#### Immunostaining: Adult MuSC sagittal hemithoraces

To image adult MuSCs in sagittal view, thoraces were fixed and bisected as previously described,^31^ with minor modification. Adult flies (<2 days) were anesthetized using CO2, and had their heads, abdomens, legs and wings removed, before fixation in 4% PFA/1% PBT for 20 minutes. Thoraces were bisected with a scalpel, followed by a further fixation for 25 minutes. Samples were washed 3x20 minutes with 0.3% PBT, and then blocked in 0.5% BSA/0.3% PBT for 30 minutes. Primary antibodies were diluted in 0.5%BSA/0.3% PBT and incubated with the sample overnight at 4 ͦC, on a rotary mixer. The following morning, samples were washed 3x20 minutes with 0.3% PBT, and then incubated with secondary antibodies diluted in 0.5%BSA/0.3% PBT for 2 hours. Secondary antibodies were removed, and samples were washed for 3x20 minutes with PBS prior to mounting on glass slides in Vectashield.

#### Quantification of jump muscle morphology

The wild-type jump muscle consists of muscle fibres arranged around a mid-line, with each fibre contacting the midline and outer surface of the muscle. In *Him* mutants, this organisation is lost, and a number of “internal fibres” arise. We counted these internal fibres, defined as fibres that do not contact the outer surface of the jump muscle, in a minimum of 6 flies per genotype, to quantify the phenotype severity.

#### Functional jumping and flight assays

Jumping ability was tested as previously reported,^21^ with minor modification. Young (<2 days) male flies were anaesthetized on a fly pad using CO_2_, and had their wings removed using dissection scissors. Flies were returned to a culture vial at 25 ͦC overnight, to recover from being anesthetized. Individual flies were transferred onto a piece of lined paper for a jumping trial, which was filmed using a smart phone. A paintbrush was used as a looming stimulus to elicit a jump escape response, by moving the brush towards the posterior of the fly. The horizontal distance travelled by the jumping fly was calculated using Tracker software (physlets.org/tracker). Each fly had 5 jumps recorded, with the mean of the longest 3 calculated and used for comparison. A minimum of 15 flies per genotype were assayed.

Flight testing was performed as previously reported,^24^ using at least 50 flies per genotype. Flies were introduced midway up a Perspex flight arena (20x20x40cm), with a light-source located above to encourage upwards flight. Flies were scored based on whether they flew upwards, horizontally, down, or not at all.

#### Quantification of wing-disc associated myoblasts and adult muscle stem cells

The wing disc-associated AMP (myoblast) pool was quantified as previously described,^32^ following commonly used methodology.^33^^,^^34^ The confocal plane in each disc that had the largest expanse of myoblasts was identified, and used to count the total number of Ebd1-positive myoblasts in that plane. Myoblast proliferation was assessed by calculating the percentage of PH3(+)/Ebd1(+) myoblasts within the larger myoblast pool. At least six imaginal discs were analysed for each genotype.

DLM-associated MuSC numbers were quantified using transverse thoracic cryosections, and were identified by their location on the periphery of muscle fibres using Zfh1 antibody staining, and by their morphology (MuSC nuclei are small). At least ten flies were analysed for each genotype and time point. For both assays the controls were *w*^*1118*^ flies.

#### Image acquisition

An Olympus BX63 with an attached Hamamatsu camera was used to image jump muscle cryosections. Images were taken using either a 4x or 10x objective, with a pE300-lite LED light source in combination with Dapi, FITC and TRITC filter cubes to visualize fluorescent probes.

Leg imaginal discs and sagittal thorax sections were imaged using a Zeiss LSM880 confocal microscope, at 20x magnification. Laser lines used included 405nm, 488nm, 561nm, 594nm and 633nm. Z-stacks were obtained with a 1μm step.

Wing imaginal discs and adult DLM cryosections were imaged using an Olympus FluoView 3000 Confocal Microscope at 20x magnification. For imaging premature differentiation in wing imaginal discs, Z-stacks were taken with a 1.04μm step. For quantification of wing imaginal disc-associated myoblasts, a single Z-plane with the largest myoblast expanse was imaged. For imaging adult DLM cryosections to quantify MuSCs, Z-stacks were taken with a 1μm step.

All image processing was performed using FIJI-ImageJ software.

### Quantification and statistical analysis

Statistical analysis was performed using either R or GraphPad Prism.

The use of parametric (ANOVA) vs non-parametric (Kruskal-Wallis) for analysis of numerical continuous data was determined by Shapiro-Wilk normality testing. Post-hoc Dunn’s was used to test for differences between groups following ANOVA, or Dunnet’s following Kruskal-Wallis.

Chi-square testing was used to compare categorical data.

Information on the statistical test used, significance level and sample size (n), is in the relevant figure legend. A convention that ∗p<0.05, ∗∗p<0.01 and ∗∗∗p<0.001 was used to display significance levels on graphs.
