# Supplementary material for: The Drosophila Him gene is essential for adult muscle function and muscle stem cell maintenance
Source: iScience. 2026 Jan 10;29(2):114670. doi: 10.1016/j.isci.2026.114670 (PMC12874454; doi:10.1016/j.isci.2026.114670)
Supplement: Document S1. Figure S1 [file mmc1.pdf]

iScience, Volume *29*

Supplemental information

The *Drosophila Him* gene is essential for adult muscle function and muscle stem cell maintenance

Robert Mitchell-Gee, Robert Hoff, Kumar Vishal, Daniel Hancock, Sam McKitrick, Cristina V. Newnes-Querejeta, Antonio Aguayo, David Liotta, Jennifer A. Waters, TyAnna L. Lovato, Richard M. Cripps, and Michael V. Taylor

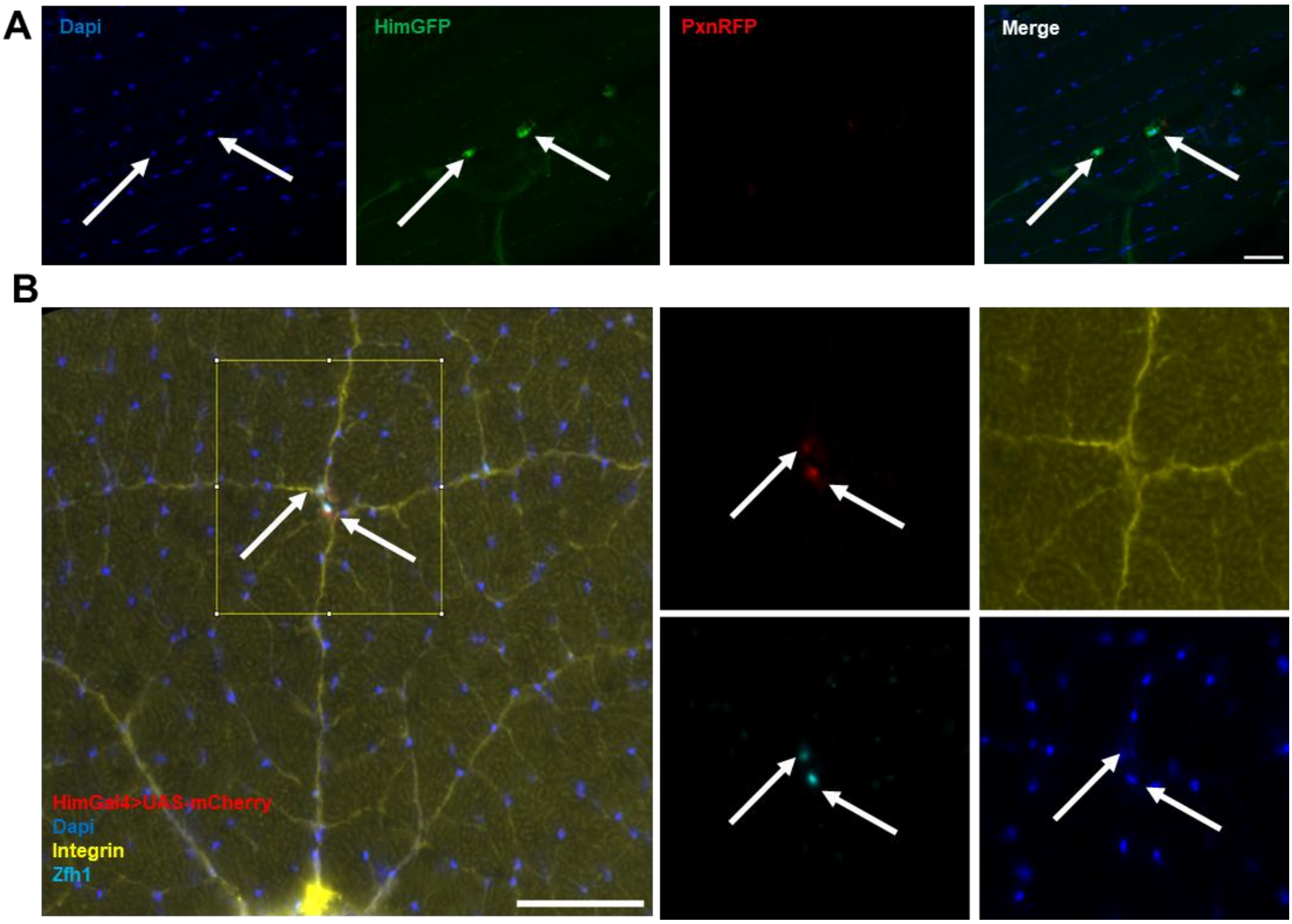

Supplemental Figure 1. HimGFP does not co-express with the hemocyte marker peroxidasin, but Him-Gal4 driven UAS-mCherry co-expresses with Zfh1 in cells at muscle fibre periphery

(A) Close up of a sagittal DLM section showing that HimGFP positive cells on a muscle fibre periphery (arrows) are negative for the hemocyte marker PxnRFP (n=3). Scale bar, 20µm. (B) A transverse cryosection showing co-localisation of Him-Gal4 driven UAS-mCherry.NLS with Zfh1 in MuSCs (arrows) on the periphery (labeled with anti-βPSintegrin) of a muscle fibre (n=3). Boxed area shown at higher magnification. Scale bar, 20µm.
